# COHORT DIFFERENCES IN PTSD SYMPTOMS AMONG VIETNAM, PERSIAN GULF, AND POST-9/11 COMBAT VETERANS

**DOI:** 10.1093/geroni/igad104.0161

**Published:** 2023-12-21

**Authors:** Maria Kurth, Dakota Witzel, Suzanne Segerstrom, Soyoung Choun, Carolyn Aldwin

**Affiliations:** Penn State University, University Park, Pennsylvania, United States; Penn State University, State College, Pennsylvania, United States; Oregon State University, Corvallis, Oregon, United States; Oregon State University, Corvallis, Oregon, United States; Oregon State University, Corvallis, Oregon, United States

## Abstract

Research examining long-term effects of military service often focuses on help-seeking Veterans who served in Vietnam, Korea, and WWII. Less is known about Veterans from more recent conflicts or community-dwelling Veterans. We administered online surveys to community-dwelling combat Veterans (N=167; Mage=57.96; 31% women) to examine cohort differences in service appraisals, post-service experiences, and predictors of PTSD symptoms. Chi-square and hierarchical regression were used to test differences in demographics, appraisals of service, post-service experiences, and PTSD symptoms. Among Veterans from Vietnam (n=60), Persian Gulf (n=68), and Post-9/11 cohorts (n=39), only age and educational attainment varied. Vietnam Veterans were older, and Persian Gulf War Veterans more likely to have a college education compared with other eras. There were no cohort differences for military appraisals or post-service experiences. In step one of hierarchical regression, men and those with lower income reported more PTSD symptoms. In step two, including cohort, Vietnam Veterans had more PTSD symptoms than the two younger cohorts, despite age now being inversely correlated with symptoms. In step three, including military and post-service experiences, demographics and cohort were no longer significantly associated with PTSD symptoms. Combat severity, undesirable appraisals, and social support were positively associated with PTSD symptoms, while desirable appraisals were inversely associated with PTSD symptoms. Cohort and demographics may be less important for Veteran mental health than service-related experiences. As Veterans of all eras move through adulthood and into later life, the importance of their miliary experiences must be considered, as it might be a ‘hidden’ variable of aging.

